# The Impact of Genetic Polymorphisms in Organic Cation Transporters on Renal Drug Disposition

**DOI:** 10.3390/ijms21186627

**Published:** 2020-09-10

**Authors:** Zulfan Zazuli, Naut J. C. B. Duin, Katja Jansen, Susanne J. H. Vijverberg, Anke H. Maitland-van der Zee, Rosalinde Masereeuw

**Affiliations:** 1 Department of Respiratory Medicine, Amsterdam UMC, University of Amsterdam, 1105 AZ Amsterdam, The Netherlands; s.j.vijverberg@amsterdamumc.nl (S.J.H.V.); a.h.maitland@amsterdamumc.nl (A.H.M.-v.d.Z.); 2 Department of Pharmacology-Clinical Pharmacy, School of Pharmacy, Bandung Institute of Technology, Jawa Barat 40132, Indonesia; 3 Division of Pharmacology, Utrecht Institute for Pharmaceutical Sciences, Utrecht University, 3584 CG Utrecht, The Netherlands; n.j.c.b.duin@students.uu.nl (N.J.C.B.D.); k.jansen@uu.nl (K.J.)

**Keywords:** organic cation transporters, drug disposition, genetic polymorphisms, kidney, drug-induced kidney injury, nephrotoxicity

## Abstract

A considerable number of drugs and/or their metabolites are excreted by the kidneys through glomerular filtration and active renal tubule secretion via transporter proteins. Uptake transporters in the proximal tubule are part of the solute carrier (SLC) superfamily, and include the organic cation transporters (OCTs). Several studies have shown that specific genetic polymorphisms in OCTs alter drug disposition and may lead to nephrotoxicity. Multiple single nucleotide polymorphisms (SNPs) have been reported for the OCT genes (*SLC22A1*, *SLC22A2* and *SLC22A3*), which can influence the proteins’ structure and expression levels and affect their transport function. A gain-in-function mutation may lead to accumulation of drugs in renal proximal tubule cells, eventually leading to nephrotoxicity. This review illustrates the impact of genetic polymorphisms in OCTs on renal drug disposition and kidney injury, the clinical significances and how to personalize therapies to minimize the risk of drug toxicity.

## 1. Introduction

The kidney is an important excretory organ for drugs and their metabolites in mammalian species, including humans. To facilitate this, in addition to filtration, the kidneys contain several transporters in their proximal tubule cells, including the solute carriers (SLCs) belonging to organic anion transporters (OATs), organic cation transporters (OCTs) and multidrug and toxic compound extrusion proteins (MATEs) and several other transporters of the ATP binding cassette (ABC) family, such as multidrug resistance proteins (MRPs) [1]. Most renally cleared drugs are excreted by multiple transporters that, in concerted action, take up molecules from the blood and efflux them into the lumen.

**Figure 1:**
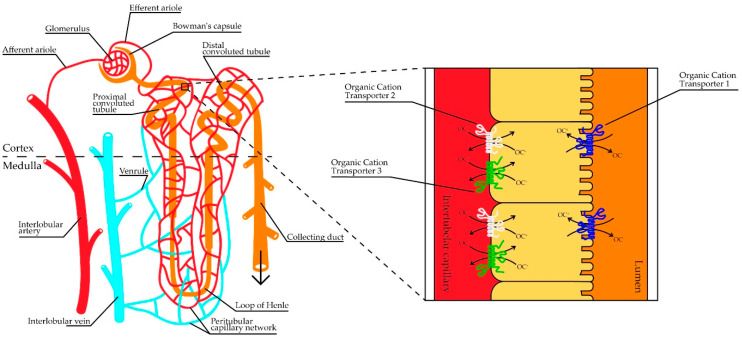
Schematic view of a nephron and a close-up of proximal tubule cells with organic cation transporters (OCTs) present in plasma membranes. In addition to glomerular filtration, organic cations (OCs) can be excreted in the proximal convoluted tubules, where OCT2 and OCT3 facilitate the uptake of compounds from the peritubular capillaries over the basolateral membrane into the intracellular space, and OCT1 mediates the uptake from the ultrafiltrate over the apical membrane.

Positively charged (cationic) drugs and drug metabolites at a physiological pH are mainly handled by OCTs (Figure 1). The OCTs facilitate the movement of endogenous and exogenous organic cationic compounds into (and from) the cell [2]. Organic cations cover a myriad of molecular structures and dimensions, which make the OCTs polyspecific transporters [2]. Multiple studies have been published on the structure and function of OCTs and at least three different subtypes have been confirmed, OCT1-OCT3 [2,3,4,5,6]. The genes encoding for OCT1 (*SLC22A1*) and OCT2 (*SLC22A2*) are clustered on the same chromosome, 2q26, whereas the gene encoding OCT3 (*SLC22A3*) is located on chromosome 6q27 [7]. All subtypes are electrogenic, facilitative transporters, independent of sodium and chloride ions and function bidirectionally [3,8]. The OCTs are jointly dependent on the electrochemical gradient caused by the cationic substrate and the membrane potential to translocate substrates [9].

Genetic variations in OCT-encoded genes might influence the functioning of OCTs and, in the end, contribute to interindividual differences in drug disposition. The advancement of genomic technologies has led to the completion of influential human genetic mapping, such as the Human Genome Project [10]. By utilizing abundance genetic information available from this project, researchers have been able to look into the genes, genetic variations and their prevalence in the population. This progress has also driven the development of the pharmacogenomics and pharmacogenetics field, resulting in the identification of genetic markers mostly in the form of single nucleotide polymorphisms (SNPs) that affect treatment efficacy and safety through their influence on drug pharmacokinetics and pharmacodynamics [11], including polymorphisms in genes that regulate transporter proteins like OCTs [12]. It is important to not only investigate the impact of genetic polymorphisms on drug effectiveness and toxicity separately, but also to understand how we can alleviate drug toxicity without compromising its effectiveness.

In this review, we will discuss the *SLC22A* family members 1–3 encoding the OCTs, their physiological roles and expressions, their known polymorphisms and how they can affect drug disposition and drug-induced kidney injury, their clinical significances and potential to personalize therapies to avoid the development of toxicity.

## 2. Role of the Organic Cation Transporters

**Figure 2:**
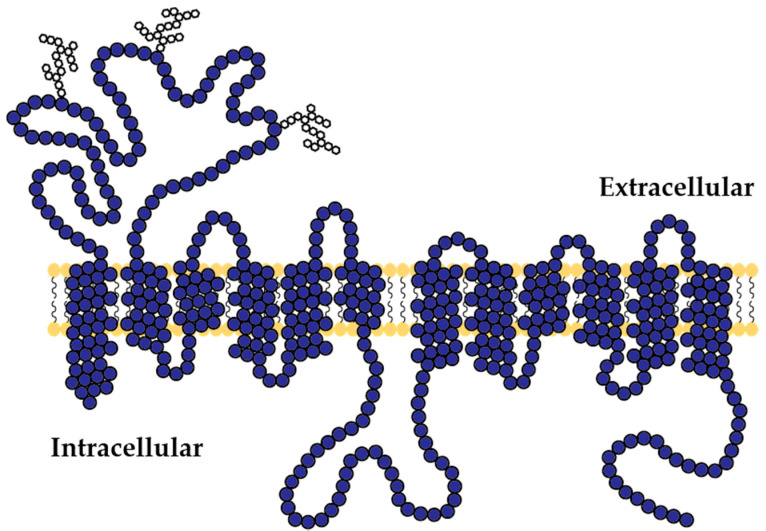
General topology of OCTs [3,13]. The proteins consist of twelve transmembranal α-helical domains. Between the first and the second loop, there is an extracellular loop that includes the N-glycolysation sites. The intracellular loop between the sixth and seventh domain includes phosphorylation sites.

The SLC22A transporter family members consist of 12 α-helical transmembrane domains with an extracellular glycosylated loop between domains 1 and 2. The large intracellular loop with the designated phosphorylation sites is found between the 6th and 7th domain [2,3,4] (Figure 2).

### 2.1. Role of OCT1

OCT1 consists of 553 amino acids. It is predominantly expressed in the liver but is also found in other tissues, which indicates that OCT1 has a housekeeping role in the body [4,14,15,16]. OCT1 is involved in the release of acetylcholine in the lungs and the placenta [17,18] and is also capable of translocating several neurotransmitters, such as serotonin and norepinephrine [2,4,14,15,18]. In the liver, OCT1 is expressed at the sinusoidal (basolateral) membrane of the hepatocytes, where it mediates the hepatic uptake of organic cations [19]. OCT1 is also expressed at the apical membrane in trachea and bronchi, in neurons where it helps to maintain the electrochemical gradient [14,18,20], in the blood–brain barrier, immune cells and the kidney [20,21]. In the human kidney, one study showed apical expression of OCT1 mediating the reabsorption of cationic drugs [20]. In contrast, another study suggested that OCT1 is localized at the basolateral membrane [22], but significant functions in the kidney have thus far only been described for rodent Oct1. Since both rOCT1 and rOCT2 were localized to the basolateral membranes of proximal tubule cells, it is hypothesized that in rodents rOct1 and rOct2 fulfill the role of OCT2 in humans [23]. As human OCT1 shows very low expression in the kidney, it is hard to specify the localization and functional relevance of the protein [16]. Differences in OCT1 expression between humans and rodents may also explain this functional variation. Basit et al. reported that OCT1 was not detected in kidneys from humans and monkeys, but was abundantly expressed in rats and mice [24].

Detailed information on OCT1 substrates, both endogenous and pharmacological compounds, can be found in Table 1.

### 2.2. Role of OCT2

OCT2 consists of 555 amino acids. It is predominantly expressed at the basolateral side of the renal proximal tubules cells, but is also found in central nervous system (CNS) tissues, such as the brain and spinal cord [3,14,16,28]. In general, OCT2 plays a role in the uptake of cationic compounds from blood to the intracellular space. Like OCT1, the expressions of OCT2 differs between humans and other species, especially in rodents, a widely used model for drug-induced nephrotoxicity screening. Expressions of OCT2 are significantly higher in rats and mice than in the human kidney cortex [24]. Pharmacological substrates that have been reported as OCT2 substrates can be found in Table 1. Several OCT1 substrates overlap with those of OCT2. In addition, OCT2 displays a decisive role in the excretion of endogenous substrates, such as creatinine [29], which is widely accepted in clinical settings to estimate glomerular filtration. Consequently, creatinine excretion, potentially inhibited by OCT2 substrates, hinders its accuracy as a kidney function marker.

In the brain, a widespread array of OCT1 substrates can bind to OCT2, including monoamine neurotransmitters, norepinephrine, serotonin, histamine and dopamine [2,30]. Yet, it has a preference for smaller hydrophilic compounds, in contrast to OCT1 that interacts more with bigger hydrophobic compounds [31]. Furthermore, OCT2 mediates the transport of anti-Parkinson’s drugs, such as amantadine and memantine through the brain–blood barrier [21]. Urakami et al. reported the existence of a splice variant of OCT2, namely OCT2A, consisting of 483 amino acids and nine transmembrane domains, which does not follow the predictable topology of the *SLC22A* family. OCT2A is suggested to have a narrower spectrum of substrates in comparison to OCT2, as it might not transport metformin [32], but it has a high affinity towards its substrates [33].

### 2.3. Role of OCT3

OCT3 consists of 556 amino acids and is widely expressed, but to a lesser extent than the other two subtypes. The transporter is predominantly found in skeletal muscles, the placenta and CNS; but is also present in the colon, the kidney, the heart and the liver [2,3,15,16,34,35,36]. OCT3 plays a role in the biliary excretion of cationic compounds in the liver and regulates the interstitial concentration of neurotransmitters in the CNS, ganglia and the heart [3,36]. The biliary excretion function is suggested to be crucial for substrates which are not transported by OCT1 or if OCT1 is inhibited by OCT1-specific substrates [21]. In the placenta, it helps the regulation of acetylcholine and the transport of organic cations [17]. OCT3 is also known as an extraneuronal monoamine transporter because of its role in the release and uptake of neurotransmitters. It plays a major role in the clearance of dopamine, norepinephrine, serotonin and histamine [36]. OCT3 is moderately expressed at the basolateral membrane of human kidney proximal tubule cells and has been regarded as less important than OCT2 [37,38], but is perceived to play a more significant role in the brain, heart and liver. In contrast with OCT1 and OCT2, the expression of OCT3 was detectable in human kidneys but not in monkeys, dogs or rodents [24]. OCT3 substrates can be found in Table 1.

## 3. Drug-Related Genetic Polymorphisms in the Organic Cation Transporter Genes

Several studies discovered polymorphisms in the OCT genes that affect the transporters’ function. These effects range from loss of transporter function and misfolded protein to a gain in transporter function. Common SNPs (minor allele frequency (MAF) ≥ 1%) in OCT genes (*SLC22A1*, *SLC22A2* and *SLC22A3*) and investigated SNPs that affect the OCTs’ function are presented in the Supplementary Materials (Supplementary Materials Figure S1) and Table 2, respectively. In addition, the following sections show that metformin, the first-line antidiabetic agent, and cisplatin, a widely used chemotherapeutic agent, are OCT substrates with the most important clinical read-outs.

Metformin is a high-affinity substrate for all OCT isoforms. Therefore, it is commonly used as a substrate prototype to investigate OCT transport activity. Metformin does not bind to plasma proteins and is excreted unchanged into the urine, which makes it a suitable candidate for studying OCTs and their genetic variations with regard to drug disposition [80,81]. To investigate the effect of genetic variations on susceptibility to drug-induced nephrotoxicity, the anti-cancer agent cisplatin is widely used due to its well-studied dose-limiting nephrotoxic effects.

### 3.1. Genetic Polymorphisms in the OCT1 Gene (SLC22A1)

*SLC22A1* is the most extensively studied OCT gene in pharmacogenetics studies. Using a candidate gene approach, several studies showed significant associations between genetic variants in *SLC22A1* and drug pharmacokinetics, although results were not always consistent. However, the genome-wide association studies carried out to date could not detect a significant association between *SLC22A1* variants and drug disposition [82,83].

Six common polymorphisms have been reported to affect the transporter function: rs34130495 (G401S), rs72552763 (M420del), rs628031 (M408V), rs6383369 (F160L), rs2282143 (P341L) and intronic rs622342. These polymorphisms are found mostly in the European and African population and are associated with reduced uptake activity of metformin [20,44,47,48,53,55,84]. Some of these polymorphisms (e.g., rs34130495, rs72552763, rs683369 and rs622342) are found, but R206C and Q97K are rare variants reported exclusively in the Asian population [48,53]. In an in vitro setting using HEK293 cells, Shu et al. showed that seven of the 12 polymorphisms of OCT1 lead to a reduced uptake of metformin, of which two polymorphisms (rs12208357 (R61C) and rs72552763 (M420del)) are common variants in Caucasians [84]. In contrast, a clinical study by Tzvetkov et al. (2009) in 103 healthy male Caucasians showed that rs12208357 (R61C), rs55918055 (C88R), rs34130495 (G401S) and rs72552763 (M420del) were associated with a significantly higher renal clearance of metformin [20]. To study these contradictory findings, Tzvetkov et al. (2009) performed a histochemical expression study and showed that OCT1 is mainly expressed at the apical membrane of renal proximal tubules and hypothesized that OCT1 plays a role in the reabsorption of metformin. This explained the higher renal clearance with reduced transport function [20]. A Danish study on 159 type 2 diabetes melitus (T2DM) patients concluded that *SLC22A1* polymorphisms decrease the steady state of metformin and are associated with a reduction in the absolute decrease in Hb1Ac [39]. However, a study in 34 healthy volunteers indicated no impact of different *SLC22A1* genotypes both on metformin steady-state pharmacokinetics and glucose utilization [85,86]. In addition, a large cohort study in 251 intolerant and 1915 fully metformin-tolerant T2DM patients in the UK showed that two reduced function OCT1 alleles were associated with metformin intolerance [87]. Furthermore, the intronic polymorphism rs622342 has been associated with reduced metformin uptake [53]. Naja et al. showed that the rs622342 variant produced higher fasting blood sugar levels and more glycosylated hemoglobin, which suggest a role in the glycemic response of metformin in type 2 diabetes [55], while Becker et al. reported a smaller glucose-lowering effect (based on HbA1c) of metformin in patients with diabetes mellitus [88]. This higher risk of reduced glycemic response was also found in rs6383369 (F160L) [50]. Although the previous study reported polymorphisms at *SLC22A1* associated with reduced metformin uptake, several clinical studies [51,89] and a meta-analyses of 5434 patients with T2DM across eight cohorts of the Metformin Genetics Consortium (MetGen) showed no significant association between *SLC22A1* polymorphisms (R61C, M420del, combined genotype for R61C and M420del–number of reduced function alleles and rs622342) and glycemic response to metformin monotherapy [89]. Therefore, while in vitro experimental studies, as well as most hypothesis-driven clinical studies (e.g., candidate gene association studies), demonstrated that *SLC22A1* polymorphisms may alter metformin disposition both in the liver and kidney and metformin intolerance, pooled analysis, like meta-analysis, indicated that these altered metformin dispositions might not be substantial enough to affect metformin effectiveness clinically.

The loss-of-function haplotypes of OCT1 were not only associated with reduced metformin transport, but also with reduced clearance of morphine [40,45,46]. OCT1 is primarily expressed in hepatocytes and the hepatic excretion of drugs will be influenced by the lower functioning haplotypes. Qiu et al. and Singh et al. showed that rs6383369 (F160L) and rs628031 (M480V) reduced the clearance of imatinib [49,52]. In OCT1, the loss-of-function genetic variants showed lower uptake activity in all reported polymorphisms for metformin and imatinib, and also showed a decreased clearance of morphine. These effects are summarized in Table 3 and discussed further in Section 4.

### 3.2. Genetic Polymorphism in the OCT2 Gene (SLC22A2)

*SLC22A2* has two common polymorphisms that affect the activity of the protein, as shown in Table 2. These polymorphisms are rs316019 (A270S) and rs596881 in the 3′ untranslated region (UTR). The variants rs316019 and rs596881 are frequently found in almost all ethnicities with MAF above 80% and 70%, respectively.

Through in silico analysis, Sajib et al. found that substrates fit better to the binding site of the A270 variant as it is more open and has a wider space than the S270 variant [56]. The polymorphism rs316019 (A270S) is associated with reduced or no changes in transport activity. Song et al. (2008) and Wang et al. (2008) both showed significantly lower activity in metformin transport with the S270 variant in healthy Korean and Chinese subjects, resulting in lower renal clearance of metformin [57,58]. A study on 1056 T2DM subjects with mostly African American ethnicity (63%) strengthens this evidence, as the minor allele rs316019 was associated with more favorable trajectories (lower disease progression) of HbA1c levels compared to the major allele carrier [90]. In contrast, a study in 23 healthy volunteers of Caucasian and African American ancestries showed that renal clearance and the net secretion of metformin were significantly higher in the variant genotype of rs316019 than in the wildtype reference genotype [91]. Finally, a meta-analysis of 5434 patients with T2DM across eight cohorts of the Metformin Genetics Consortium (MetGen) showed no statistically significant association between rs316019 polymorphisms and glycemic response to metformin monotherapy [89].

A recent in vitro study using a 3-[4,5-dimethylthiazol-2-yl]-2,5 diphenyl tetrazolium bromide (MTT) assay showed that decreased expression of hOCT2 A270S resulted in protection against cisplatin cellular toxicity compared to hOCT2 wildtype cells [59]. However, creatinine displayed a higher affinity for hOCT2 A270S, resulting in higher serum creatinine clearance in A270S compared to wildtype, which suggested an increased function [59]. In addition, the reported associations between rs316019 polymorphisms and cisplatin-induced nephrotoxicity are also conflicting among clinical studies using serum creatinine as a renal function parameter. This is possibly due to the influence of age, sex, ethnicity and the nature of creatinine itself as an OCT2 substrate [97]. Three studies (a study on 80 Dutch patients [60] and two Japanese studies consisting of 31 children [92] and 53 adults [93]) reported that individuals carrying a variant of rs316019 less frequently experienced creatinine-based cisplatin nephrotoxicity compared to individuals with the wildtype genotype. A study in 123 Chinese patients also displayed lower changes of cystatin C in patients with a mutant genotype [94]. However, a study in 95 Japanese esophageal cancer patients reported no association [98]. Moreover, a study in 206 patients (92% Caucasians) even showed that patients who carried a variant genotype had higher levels of KIM-1, a novel biomarker of kidney injury, compared to wildtype carriers [66]. These findings were also confirmed by a study in 159 Canadian subjects that reported a higher risk of creatinine-based cisplatin nephrotoxicity in patients bearing the variant allele compared to the wildtype allele [61]. A study on 403 Chinese non-small cell lung cancer (NSCLC) patients displayed that rs316019 was associated with lower risk of hepato- and hematotoxicity in platinum-based chemotherapy [95]. Furthermore, this polymorphism was also associated with a lower risk of ototoxicity both in adult and pediatric patients treated with cisplatin according to a German study [96].

The intronic rs596881 polymorphism was shown to have a renoprotective effect as the estimated glomerular filtration rate (eGFR, a combination of serum creatinine-, age- and sex-based renal function estimation) was preserved in the rs596881 haplotypes [66].

The less common polymorphisms, rs8177516 (R400S), rs8177517 (K432Q), rs8177507 (M165I), rs201919874 (T199I) and rs14540955 (T201M), all showed reduced transporter activity [44,47,57,62,63,65,79]. Moeez et al. (2019a) investigated the T199I variant in which threonine199 is changed to isoleucine199, which alters the protein structure by acquiring a catalytic residue, losing the loop and glycosylation site. Furthermore, it gains an α-helix structure and a molecular recognition feature. These changes affect the binding pocket of OCT2 and reduces the transporter’s activity [65]. Leabman et al. (2002) showed that rs8177516 (R400C) and rs8177507 (M165I) had reduced dose–response curves of 1-methyl-4-phenylpyridinium (MPP^+^) than the wildtype, by saturating OCT2 with this substrate. Of these three variants, the rs8177516 (R400C) variant had the lowest activity. The polymorphisms rs316019 (A270S) and rs8177517 (K432Q) had similar dose responses to a saturating amount of MPP^+^ when compared to wildtype. They also studied the effect of inhibiting compounds on the transporter variants, rs8177516 (R400S), rs8177517 (K432Q), rs8177507 (M165I) and rs316019 (A270S), and suggested that tetrabutylammonium is the more potent inhibitor for the rs8177516 (R400S) and rs8177517 (K432Q) variants, whereas rs316019 (A270S) showed decreased inhibition by tetrabutylammonium [64]. Song et al. (2008) and Choi et al. (2012) showed that rs201919874 (T199I) and rs14540955 (T201M) variants have a lower renal clearance of metformin and consequentially a higher plasma concentration [58,63]. Choi et al. (2012) also showed a lower renal clearance for MPP^+^ and lamivudine in rs201919874 (T199I), rs14540955 (T201M) and rs316019 (A270S) variants [63]. Furthermore, Choi et al. (2013) showed that only the homozygous rs14540955 (T201M) had a significantly lower lamivudine clearance [68]. Kashi et al. (2015) suggested that rs14540955 (T201M) changes resistance to insulin, as the study showed an increase in the Homeostatic Model Assessment for Insulin Resistance (HOMA-IR) in this variant. This is probably due to the reduced transport of metformin [62].

### 3.3. Genetic Polymorphisms in the OCT3 Gene (SLC22A3)

*SLC22A3* has four common polymorphisms found in European, African, Asian, East Asian, South Asian, Caribbean and Native American, Latin American and Hispanic populations, as shown in Table 2. The polymorphisms include: rs2292334 (A411), rs3088442 in the 3′ UTR, rs555754 in the 5′ UTR and intronic rs376563. These polymorphisms account for more than 10% of the MAF in the ethnicities described above. Other non-synonymous polymorphisms have a MAF of ≤ 1%.

The rs8187717 (A116S), rs8187725 (T400I), rs1221246 (A439V) and rs8187722 (L346) variants are all associated with a reduced uptake of metformin [72,73,74] as well as catecholamines and histamine [69,73,74]. The latter substrates are also affected by the polymorphisms rs9365165 (G475S) and V423F. Chen et al. (2010) investigated several polymorphisms, of which only the rs8187715 (T44M) variant showed enhanced uptake activity of OCT3 [72]. Hakooz et al. (2017) investigated the synonymous polymorphisms rs2292334 (A411) and rs8187722 (L346) in which the heterozygous variant rs2292334 showed a higher plasma concentration and lower clearance for metformin compared to the wildtype. On the other hand, the rs8187722 (L346) polymorphism showed no significant reduction in metformin clearance [69]. Analysis of pharmacodynamic data in 57 healthy volunteers with mixed ethnicities (majority African American, *n* = 33; Asian, *n* = 18; Caucasian, *n* = 6) showed that the variant rs2076828 was associated with reduced response to metformin during an oral glucose tolerance test [77]. Furthermore, a study in 233 newly diagnosed Caucasian T2DM patients showed that minor alleles of rs2481030 located in the intergenic region between *SLC22A2* and *SLC22A3* are associated with metformin inefficiency [78]. However, another study in 103 healthy male Caucasians reported no significant effect of several *SLC22A3* variants in the disposition to metformin [20].

Besides polymorphisms affecting uptake activity, a polymorphism acting as genetic marker was found. Mahrooz et al. (2017) and Moeez et al. (2019b) investigated the polymorphism rs3088442 in the 3′ UTR of the *SLC22A3* gene. They hypothesized that this polymorphism could be a genetic marker for an increased risk of type 2 diabetes, but the study showed a protective effect on the susceptibility to type 2 diabetes. The minor A allele was shown to have a positive effect, in contrast to the major G allele showing a negative effect on the metformin response [70,71]. Furthermore, Chen et al. (2013) suggested that the polymorphisms rs555754 and rs60515630, both in the 5′ upstream region, are involved in the transcription rate of the *SLC22A3* gene. The rs555754 and rs60515630 variants showed a higher transcription rate of *SLC22A3* and a higher expression of OCT3 in the liver [75]. Furthermore, OCT3 has a low expression in prostate cancer lines and higher expression levels of OCT3 have been associated with cancer suppressive effects, possibly due to the enhanced transcription rate and higher expression caused by the rs555754 and rs60515630 polymorphisms [75]. It has been suggested that OCT3 could be a candidate genetic biomarker to predict therapy effectiveness in various diseases, especially cancer [4,36,75].

Previously, an epidemiological study highlighted the differences in metformin response between various self-reported ethnic origins, in which patients with an African American background appear to have a better glycemic response to metformin than European American patients [99]. One might hypothesize that differences in allele distributions of pharmacogenomic variants among various ethnicities could be associated with variations in metformin response. In general, individuals with an African background have lower MAF in metformin-related OCT variants than European individuals, as observed in rs628031 (Table 2). However, there is limited evidence that racial or ethnic variations account for differences in metformin response to date. Furthermore, most of the pharmacogenetic research in metformin has been focused on European and Asian individuals. Further research will be needed to characterize the response to metformin in pharmacogenomic variants across ethnicities, especially African.

## 4. Impact of Pharmacogenetic Variants in OCTs in Precision Medicine

Overall, the antidiabetic drug metformin and antineoplastic drug cisplatin are the most extensively studied drugs related to pharmacogenetic variants in OCTs. Based on in vitro, in vivo and clinical pharmacogenetic studies in *SLC22A1-3* genes, metformin is the most comprehensively studied drug, covering pharmacokinetics and drug response outcomes due to its high affinity to all three OCT subtypes. Pharmacogenetic variants in OCTs related to cisplatin are also widely studied, especially variants in *SLC22A2* since cellular cisplatin uptake is mainly regulated by OCT2. Published studies have covered both the efficacy and adverse drug reaction aspects of cisplatin. The impacts of *SLC22A3* pharmacogenetic variants are still insufficiently unraveled, especially in clinical settings. More information on how OCT polymorphisms affect OCT substrates is presented in Table 3.

Metformin is considered to be the first-line antidiabetic drug to treat T2DM and has been used for more than 60 years. Metformin itself is perceived as the safest antidiabetic agent in chronic kidney disease. In addition, independent of its hypoglycemic effect, it reduces the risk of myocardial infarction, stroke and mortality in patients with T2DM and chronic kidney disease (CKD) [100]. However, its use has been limited in severe renal impairment patients because of a higher risk of lactic acidosis [100,101]. Apart from that, the clinical utility of *SLC22A1* and *SLC22A2* variants to assist the precision medicine of metformin is questionable, as a meta-analysis of 5434 patients with T2DM across eight cohorts of the Metformin Genetics Consortium (MetGen) showed no significant association between *SLC22A1* (R61C, M420del, combined genotype for R61C and M420del–number of reduced function alleles and rs622342) and *SLC22A2* polymorphisms (rs316019) and glycemic response to metformin monotherapy [89], and no organic cation transporter variants were found to be associated with metformin disposition through genome-wide studies. Through a three-stage genome-wide association study (GWAS) in 10,577 subjects of European ancestry, the MetGen Consortium reported that rs8192675 in the intron of *SLC2A2*, which encodes the facilitated glucose transporter GLUT2, was associated with a greater reduction in HbA1c [82]. A GWAS on 1312 white and black participants in the ACCORD trial showed that common and rare variants in *PRPF31*, *CPA6* and *STAT3* were associated with metformin response [83]. In addition, a recent systematic review suggested that the role of *SLC22A1* variants in individual responses to metformin is population-specific due to high heterogeneity among studied populations [102]. However, the combined effect of the *SLC22A1* genotype is valuable to predict metformin intolerance [87,103].

Cisplatin is arguably one of the most studied nephrotoxic drugs. It is a highly potent chemotherapeutic agent, but its therapeutic use is limited due to the development of nephrotoxicity and ototoxicity. Cytotoxic events include oxidative stress, cytoplasmic organelle dysfunction (endoplasmic reticulum stress and mitochondrial dysfunction), DNA damage and activation of apoptotic pathways (death receptor and caspase-dependent pathway) [104]. This leads to cell necrosis. OCTs have been proven to play an important role in cisplatin nephrotoxicity. When cisplatin is taken up by a basolateral transporter, predominantly OCT2, but also the copper transport protein (copper transporter receptor 1, CTR1), but not excreted as fast or at all by apical transporters, such as the multidrug and toxic compound extrusion proteins (MATEs), it will accumulate inside the cell and affect multiple cell functions, eventually resulting in cell death [104]. As mentioned before, rs316019 (A270S) is shown to modify the nephrotoxicity of cisplatin, although the result was not consistent [43,60]. Instead of OCT variants, a GWAS on 1010 testicular cancer survivors reported that rs1377817 of *MYH14* was associated with the serum platinum residuals [105]. Besides nephrotoxicity, ototoxicity is another unwanted effect of cisplatin that is extensively studied. However, none of the OCT genetic variants was proven to be associated with cisplatin-induced ototoxicity. Through candidate gene studies, rs9332377 *COMT* [106,107,108] and rs12201199 *TPMT* [106,109,110] were associated with ototoxicity, although the direction of association is not consistent among studies. Meanwhile, the rs4788863 *SLC16A5* variant demonstrated an otoprotective effect [111,112]. Two GWASs reported SNPs that are associated with an increased risk of ototoxicity: rs1872328 in *ACYP2* [113] and rs62283056 in *WFS1* [114]. The clinical evidence for those SNPs was also supported with functional validation studies [115].

The development of genomic technologies has allowed the unbiased investigation of genetic variation across the genome, like GWASs. GWASs on metformin are a good example on how such an approach may reveal new and relevant variants through observed outcomes. In cisplatin nephrotoxicity, however, differences in outcome definition were proven to contribute to inconsistent associations between the genetic variant and the outcome [61]. Thus, such an effort should be accompanied by more robust kidney injury biomarkers than serum creatinine for a better phenotyping, such as kidney injury molecule-1 (KIM-1), β2-microglobulin (B2M), cystatin C, clusterin and trefoil factor-3 (TFF-3), to define nephrotoxicity [116]. The selection of more sensitive and specific drug-induced kidney injury biomarkers will be a feasible solution for the creatinine limitations we mentioned earlier. However, it should be noted that the large sample size of specific ethnicity populations and similar clinical characteristics of the population required to detect a significant genome-wide association might be a major challenge, as demonstrated by the metformin GWAS. Alternatively, functional validations in in vitro settings using gene-editing techniques such as CRISPR-Cas9 and pharmacokinetic validation of current associated SNPs may lead to robust evidence on the mechanistic role of the associated SNPs on cisplatin disposition. Moreover, multilayer omics profiling, such as genomics, epigenomics, transcriptomics, metabolomics and proteomics, observed in the same subject would generate valuable knowledge to reveal the whole mechanism of action, how drugs affect the body’s physiological processes and demonstrate their efficacy and toxicity, especially for metformin and cisplatin. Such comprehensive information would be a significant step to precision therapy of metformin and cisplatin to reduce their toxicity and optimize their effectiveness at an individual level. Finally, a clinical study on genotype-guided prescribing would also offer an answer on how utilizing individual genetic information is clinically significant in metformin and cisplatin therapy.

## 5. Conclusions

The current evidence and literature show several promising genetic biomarkers in the prediction of OCT drug substrate disposition, especially metformin and cisplatin. Meanwhile, the evidence on OCT genetic variants’ influence on renal drug disposition remains inconsistent, especially for *SLC22A2* in cisplatin. Therefore, in addition to current findings, data from larger cohorts with multiomics approaches whenever possible, along with the necessary functional validation, would be significantly beneficial to explain comprehensively interindividual variability in the pharmacokinetic, pharmacodynamic, effectiveness and toxicity profiles of drugs, including nephrotoxicity. Finally, these data could drive the precision therapy of drugs: avoiding and minimizing unwanted effects and enhancing drug effectiveness simultaneously.

## Figures and Tables

**Table 1:** Organic cation transporters and their endogenous and drug substrates.

Type of OCT	Endogenous Substrates	Drug Substrates			References
OCT1	monoamine neurotransmitters ** norepinephrine ** serotonin ** histamine ** dopamine **	Acyclovir albuterol * amiloride * amisulpride ** atenolol * atropine * barberine ** cimetidine * clidinium daunorubicin debrisoquine diltiazem evafirenz fenoterol *	furamidine ganciclovir ipratropium * irinotecan lamivudine ** lamotrigine metformin ** metoclopramide * oxaliplatin ** paclitacel pentamidine picoplatin * procainamide *	Ranitidine * salbutamol selegiline sumatriptan * terazosin * terbutaline * tiotropium * triamterene * trimethoprim * tropisetron varenicline * zalcitabine	[8,21,25,26]
OCT2	creatinine monoamine neurotransmitters ** norepinephrine ** serotonin ** histamine ** dopamine **	Albuterol * amantadine amiloride * amiodarone amisulpride ** atenolol * atropine * barberine ** cimetidine * cisplatin famotidine fenoterol *	ganciclovir ifosfamide ipratropium * lamivudine ** memantine metformin ** metoclopramide * oxaliplatin ** picoplatin * procainamide *	Ranitidine * Sumatriptan * Terazosin * Terbutaline * Tiotropium * Triamterene * Trimethoprim * Varenicline * zalcitabine zidovudine	[1,21,27]
OCT3	monoamine neurotransmitters ** dopamine ** norepinephrine ** serotonin ** histamine **	Amisulpride ** berberine ** cisplatin etilefrine	Lamuvidine ** lidocaine metformin **	pramipexole oxaliplatin ** quinidine	[8,21]

* Substrate of both OCT1 and OCT2. ** Substrate of OCT1, OCT2 and OCT3.

**Table 2:** Studied OCT1-3 polymorphisms with minor allele frequency (MAF).

Gene	Polymorphism	AA *	Allele Change	Annotation (DNA Strand **)	Alleles		MAF per Ethnicity #								References
					Minor	Major	EU	Af	As	EA	SA	C	LA	Other	
*SLC22A1* (OCT1)	rs12208357	R61C	181C>T	Coding (plus)	T	C	0.0723	0.0281	0	0	0.04	0.037	0.0216	0.0701	[12,20,39,40,41,42,43]
	rs55918055	C88R	262T>C	Coding (plus)	C	T	0.00303	0.006	0	0	0	0	0	0	[12,44,45]
	rs34130495	G401S	1201G>A	Coding (plus)	A	G	0.0261	0.005	0	0	0	0.02	0.0127	0.0197	[39,45,46]
	rs72552763	M420del	1260-1262delGAT	Coding (plus)	del	GAT	0.1148	0.053	0	0	0	0	0	0.0805	[12,20,39,40,41,43,46]
	rs34059508	G465R	1393G>A	Coding (plus)	A	G	0.02274	0.008	0	0	0	0	0	0.0194	[12,20,39,40,43,45,47]
	rs628031	M408V	1222A>G	Coding (plus)	A	G	0.402712	0.2663	0.3	0.308	0.3666	N/D	0.2058	0.3701	[20,40,43,48,49]
	rs683369	F160L	480G>C	Coding (plus)	G	C	0.1977	0.123	0.22	0.36	0.2	N/D ***	0	0.1712	[48,50,51,52]
	N/A ****	R206C	616C>T	Coding (plus)	T	C	N/D	N/D	0.008 ^1^	N/D	N/D	N/D	N/D	N/D	[48]
	N/A	Q97K	289C>A	Coding (plus)	A	C	N/D	N/D	0.017 ^1^	N/D	N/D	N/D	N/D	N/D	[48]
	rs200684404	P117L	350C>T	Coding (plus)	T	C	0.00008	0.001	0.012	0.018	0	N/D	0	0.0004	[48]
	rs34447885	S14F	41C>T	Coding (plus)	T	C	0.00033	0.012	0	0	0	N/D	0	0.0011	[20,40,53]
	rs36103319	G220V	659G>T	Coding (plus)	T	G	0.00013	0	0	0	N/D	N/D	N/D	0	[44]
	rs34104736	S189L	566C>T	Coding (plus)	T	C	0.00152	0	0	0	N/D	N/D	N/D	0.0014	[47,53,54]
	rs2282143	P341L	1022C>T	Coding (plus)	T	C	0.014788	0.0621	0.094	0.086	0.08	0.069	0.0384	0.0181	[47,49,53]
	rs622342	N/A	C>A	Intron 9	C	A	0.363843	0.1871	0.192	0.155	0.2764	N/D	0.3689	0.3048	[53,55]
*SLC22A2* (OCT2)	rs316019	A270S	808A>C	Coding (minus)	A	C	0.103508	0.1541	0.11	0.11	0.1156	N//D	0.0533	0.10206	[20,43,56,57,58,59,60,61,62,63]
	rs8177516	R400S	1198G>T	Coding (minus)	T	G	0.000205	0.0121	0	0	0.0002	0.026	N/D	0.0014	[44,47]
	rs8177517	K432Q	1294A>G	Coding (minus)	G	A	0.001096	0.0243	0	0	0.0022	0.003	N/D	0.0022	[44,47]
	rs8177507	M165I	495G>A	Coding (minus)	A	G	0.000009	0.0062	0	0	0	0	N/D	0.0002	[44,64]
	rs201919874	T199I	596C>T	Coding (minus)	T	C	0 ^2^	0^2^	0^2^	0.0007 ^2^	0 ^2^	N/D	N/D	N/D	[63,65]
	rs596881	N/A	T>C	3′UTR (plus)	T	C	0.101541	0.2983	0.104	0.102	0.1294	0.136	0.0707	0.1224	[66,67]
	rs145450955	T201M	602C>T	Coding (minus)	T	C	0.00001	0	0.045	0	N/D	N/D	N/D	0.0001	[57,58,62,68]
*SLC22A3* (OCT3)	rs2292334	A411	1233G>A	Coding (plus)	A	G	0.364435	0.1458	0.448	0.403	0.2702	0.331	0.5086	0.3458	[69,70,71]
	rs8187715	T44M	131C>T	Coding (plus)	T	T	0.006 ^4^	0.006 ^3^	0.012 ^3^	N/D	N/D	N/D	N/D	0.0009	[72]
	rs8187717	A116S	346G>T	Coding (plus)	T	G	0	0.0017 ^4^	0	0	0	0	0	0	[72]
	rs8187725	T400I	1199C>T	Coding (plus)	T	C	0.00005	0	0	0	0	0	0	0	[72,73,74]
	rs12212246	A439V	1316C>T	Coding (plus)	T	C	0.00001	0	0	0	0	0	0	0	[73,74]
	rs9365165	G475S	1423G>A	Coding (plus)	A	G	0.00013	0	0	0	0	0	0	0	[73,74]
	rs8187722	L346	1038A>G	Coding (plus)	G	A	0.001233	0.0338	0	0	0.0002	0	0	0.0079	[69,72]
	N/A	V423F	1267G>T	Coding (plus)	T	G	0 ^4^	0 ^4^	N/D	0.068 ^4^	N/D	N/D	N/D	N/D	[72]
	rs3088442	N/A	564G>A	3′UTR (plus)	A	G	0.36755	0.08969	0.35	0.35	0.08	0.5	0.92	0.335	[70,71]
	rs555754	N/A	−29G>A	5′UTR (plus)	A	G	0.46766	0.5441	0.25	0.21	0.33	0.515	0.3764	0.477	[75,76]
	rs60515630	N/A	−81G>delAG	Upstream	G	del	0.0029	0.1	N/D	N/D	N/D	N/D	N/D	N/D	[75,76]
	rs376563	N/A	976-6046T>C	Intron 5 (plus)	T	C	0.47480	0.2523	0.35	0.386	0.47	0.444	0.3652	0.4119	[67]
	rs2076828	N/A	698C>G	3′UTR (plus)	G	C	0.4249	0.46	0.8	0.5377	0.8	N/D	N/D	0.4	[77]
	rs2481030	N/A	A>G	Intergenic	G	A	0.3404	0.177	0.17	0.17	0.81	N/D	0.2671	0.306	[78]

* AA: Amino acid. ** Plus and minus signs mean on which DNA strand the polymorphisms is found. *** N/D: No data. **** N/A: Not available. # Frequency database used is ALFA (Allele Frequency Aggregator) if not otherwise mentioned. EU is European population; Af is African population; As is Asian population; EA is East Asian population; SA is South Asian population; C is Caribbean and Native American Population; LA is Latin American and Hispanic population; Other is small non-designated populations. ^1^ Frequency found in Chen (2010a) [48]. ^2^ Frequency was found in Kang et al. (2007) [79]. ^3^ Frequencies from HapMap project. ^4^ Frequencies found in Chen (2010b) [72].

**Table 3:** Organic cation transporter function and substrates affected by polymorphisms.

Gene	SNP	Ref	Drugs and Chemicals	Effect
*SLC22A1* (OCT1)	rs12208357	[20,39,41,42,43,87]	Metformin, morphine	Reduced uptake activity; decrease in steady-state concentration of metformin; associated with metformin intolerance
	rs55918055	[44,45]	Metformin	Reduced uptake activity
	rs34130495	[39,45,46,87]	Metformin, * MPP+	Reduced uptake activity; decrease in steady-state concentration of metformin; associated with metformin intolerance
	rs72552763	[20,39,40,41,42,43,46,87]	Metformin, morphine, MPP+	Reduced uptake activity and decreased morphine clearance; decrease in steady-state concentration of metformin; associated with metformin intolerance
	rs34059508	[20,39,43,45,47,87]	Metformin, MPP+	Reduced uptake activity; decrease in steady-state concentration of metformin; associated with metformin intolerance
	rs628031	[20,43,48,49]	Metformin, imatinib	Reduced imatinib clearance
	rs683369	[48,50,51,52]	Metformin, imatinib	Reduced function and reduced imatinib clearance
	R206C	[48]	Metformin	Reduced uptake activity, reduced function
	Q97K	[48]	Metformin	Reduced uptake activity
	rs200684404	[48]	Metformin	Reduced uptake activity
	rs34447885	[20,53]	Metformin	Reduced uptake activity
	rs36103319	[44]	Metformin	Reduced uptake activity
	rs34104736	[47,53,54]	Metformin	Reduced uptake activity
	rs2282143	[47,49,53]	MPP+	Reduced uptake activity
	rs622342	[39,53,55,88]	Metformin	Reduced uptake activity; decrease in steady-state concentration of metformin; smaller HbA1c lowering effect
*SLC22A2* (OCT2)	rs316019	[20,43,56,57,58,59,60,61,62,63,66,90,91,92,93,94,95,96]	Metformin, cisplatin, creatinine, MPP+, lamivudine	Reduced uptake activity, lower renal clearance of metformin, higher renal clearance of metformin, lower HbA1c levels in metformin users, lower nephrotoxicity, higher nephrotoxicity, lower hematotoxicity, lower hepatotoxicity
	rs8177516	[44,47]	Metformin, MPP+, ** TBA	Reduced uptake activity
	rs8177517	[44,47]	Metformin, MPP+, TBA	Reduced uptake activity
	rs8177507	[44,64]	Metformin	Reduced uptake activity
	rs201919874	[63,65]	Metformin, MPP+, lamivudine	Damaged protein, reduced activity
	rs596881	[66,67]	N/A	Renoprotective effect and maintenance of eGFR, hypertension
	rs145450955	[57,58,62,68]	Metformin, MPP+, lamivudine, insulin	Reduced activity, changed insulin resistance
*SLCC22A3* (OCT3)	rs2292334	[69,70,71]	Metformin	Reduced activity
	rs8187715	[72]	Metformin, * MPP+, *** catecholamines	Enhanced uptake activity
	rs8187717	[72]	Catecholamines, metformin, MPP+, histamine	Reduced uptake activity
	rs8187725	[72,73]	Catecholamines, metformin, MPP+, histamine	Reduced uptake activity
	rs12212246	[73]	Catecholamines, metformin, MPP+, histamine	Reduced uptake activity
	rs9365165	[73]	Histamine	Reduced histamine uptake
	rs8187722	[69,72]	Metformin	Reduced uptake activity
	V423F	[72]	Catecholamines	Reduced uptake activity
	rs3088442	[70,71]	Metformin	Genetic risk marker for T2DM #, A allele has protective effect
	rs555754	[75]	N/A	Higher transcription rate, higher expression
	rs60515630	[75]	N/A	Higher transcription rate, higher expression
	rs376563	[67]	N/A	Effect on diabetic nephropathy and hypertension
	rs2076828	[77]	Metformin	Reduced response to metformin
	rs2481030	[78]	Metformin	Metformin inefficiency

* MPP+: 1-methyl-4-phenylpyridinium. ** TBA: tetrabutylammonium. *** Catecholamines: serotonin, norepinephrine, acetylcholine and dopamine. # T2DM: type 2 diabetes.

## Supplementary Materials

The following are available online at https://www.mdpi.com/1422-0067/21/18/6627/s1, Figure S1: Common SNPs (MAF≥1%) in *SLC22A1*, *SLC22A2* and *SLC22A3* (Extracted from UCSC Genome Browser on Human).

## Abbreviations

SLC	Solute carrier
OAT	Organic anion transporters
OCT	Organic cation transporter
SNP	Single nucleotide polymorphism
MATE	Multidrug and toxic compound extrusion
CTR	Copper transporter
ABC	ATP-binding cassette
MRP	Multidrug resistance protein
OC	Organic cation
CNS	Central nervous system
MAF	Minor allele frequency
HEK293	Human embryonic kidney 293 cells
T2DM	Type 2 diabetes mellitus
eGFR	Estimated glomerular filtration rate
MPP+	1-methyl-4-phenylpyridinium
TBA	Tetrabutylammonium
HOMA-IR	Homeostatic model assessment for insulin resistance
3′UTR	Three prime untranslated region
MTT	3-[4,5-dimethylthiazol-2-yl]-2,5 diphenyl tetrazolium bromide
KIM-1	Kidney injury molecule-1
B2M	β2-microglobulin
TFF-3	Trefoil factor-3
NSCLC	Non-small cell lung cancer
CKD	Chronic kidney disease
GWAS	Genome-wide association studies
CRISPR-Cas9	Clustered Regularly Interspaced Short Palindromic Repeats and Cas genes
