# Supplementary figures and images for: The Impact of Genetic Polymorphisms in Organic Cation Transporters on Renal Drug Disposition
Source: Int J Mol Sci. 2020 Sep 10;21(18):6627. doi: 10.3390/ijms21186627 (PMC7554776; doi:10.3390/ijms21186627)

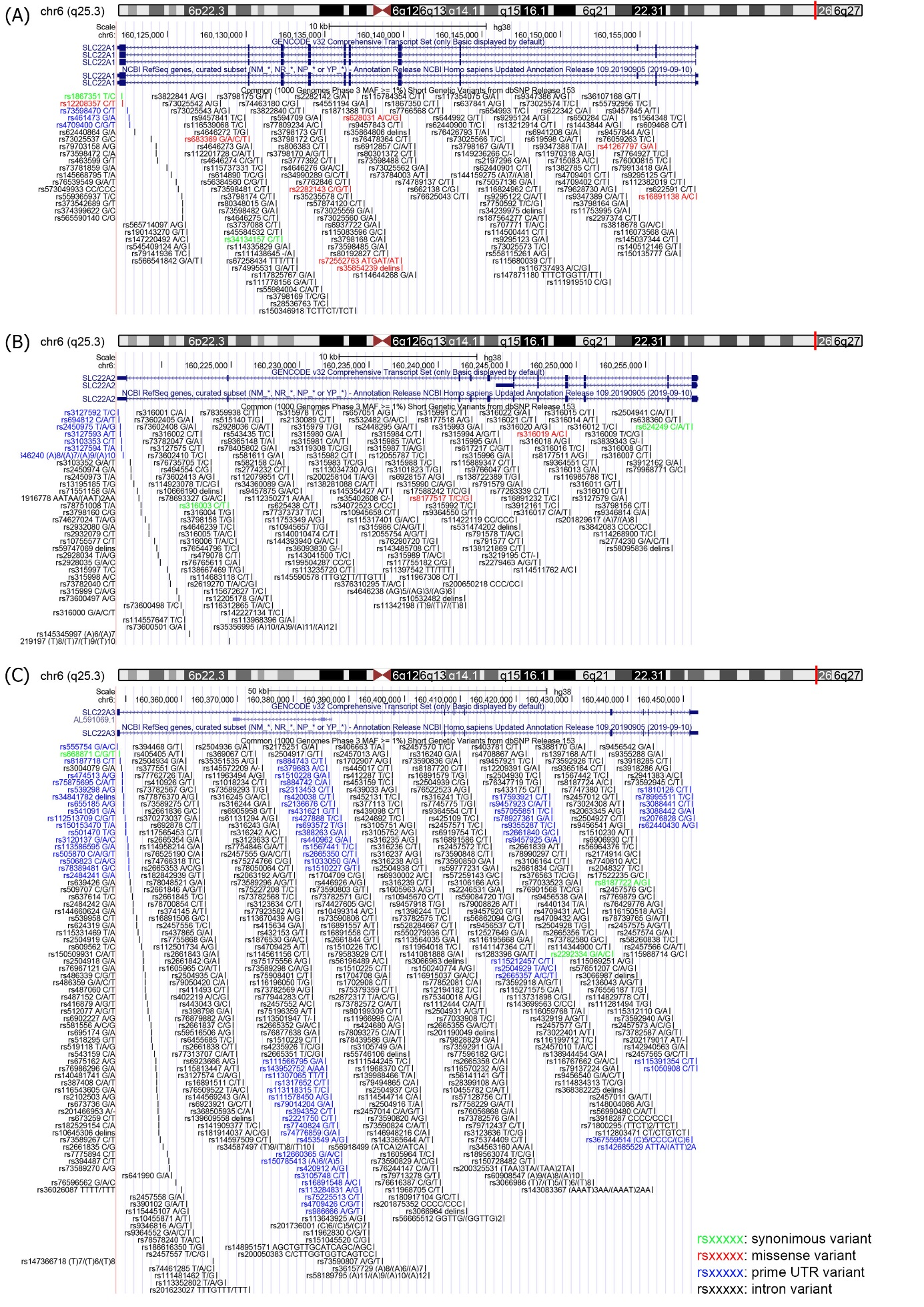

Supplement: Supplementary file 1 [file ijms-21-06627-s001.zip › Fig S1. Common SNPs.png]
